# Association between breastfeeding duration and educational attainment in rural Southwest Uganda: a population-based cohort study

**DOI:** 10.1080/16549716.2024.2338023

**Published:** 2024-04-23

**Authors:** Shamsudeen Mohammed, Clara Calvert, Joseph O Mugisha, Makanga Ronald, Gershim Asiki, Judith R Glynn, Laura L Oakley, Milly Marston

**Affiliations:** a Department of Non-communicable Disease Epidemiology, Faculty of Epidemiology and Population Health, London School of Hygiene & Tropical Medicine, London, UK; b Centre for Global Health, Usher Institute, University of Edinburgh, Edinburgh, UK; c Department of Population Health, Faculty of Epidemiology and Population Health, London School of Hygiene & Tropical Medicine, London, UK; d MRC/UVRI and LSHTM Uganda Research Unit, Entebbe, Uganda; e African Population and Health Research Center (APHRC), Nairobi, Uganda; f Department of Infectious Disease Epidemiology, Faculty of Epidemiology and Population Health, London School of Hygiene & Tropical Medicine, London, UK; g Centre for Fertility and Health, Norwegian Institute of Public Health, Oslo, Norway

**Keywords:** Breastfeeding, educational attainment, schooling, educational achievement, Uganda, sub-Saharan Africa

## Abstract

**Background:**

Breastfeeding is important for early childhood nutrition and health. The positive effects on educational outcomes may be attributed to socioeconomic factors. Socioeconomic status is not a strong predictor of breastfeeding in sub-Saharan African countries. Yet, few studies have investigated the association between breastfeeding and educational outcomes in these countries.

**Objective:**

This study investigated the association between breastfeeding duration and children’s educational attainment in rural Southwest Uganda.

**Methods:**

We analysed longitudinal data on 3018 children who had information on breastfeeding and were followed for at least 5 years, with at least one primary school grade recorded by 2005. Data on breastfeeding duration were collected from mothers. The highest school grade was recorded repeatedly between ages 6 and 12 years. We calculated age-for-grade based on whether a child was on, over, or under the official age for a grade. Generalised estimating equations and binary logistic regression estimated the effect of breastfeeding duration on being 2 years, 3 or more years, or any years over-age for grade in primary school, adjusting for socioeconomic status and maternal-child characteristics.

**Results:**

Most mothers breastfed for more than a year. Just over one-third breastfed for 18–23 months, and 30% breastfed for longer. By age eight, 42% of the children were two years over-age for their grade. Three or more years over-age for grade increased from 19% at age nine to 56% at age 12. Both adjusted and unadjusted estimates were consistent in showing reduced odds for children being 2 years, 3 or more years, or any years over-age for grade among children breastfed for 7–12, 13–17, 18–23, and > 23 months compared to those breastfed for 0–6 months. There was no evidence to support an overall association between breastfeeding duration and being over-age for grade. There was no evidence of association in the sex and age sub-group analyses.

**Conclusion:**

Although we found no association between breastfeeding duration and educational attainment, breastfeeding remains important for children’s health and nutrition, and mothers should be encouraged and supported to breastfeed for the recommended duration.

## Paper context

- **Main findings:** We found no clear evidence of an association between breastfeeding duration and educational attainment in rural Uganda.
- **Added knowledge:** The findings of this study contribute to a better understanding of the relationship between breastfeeding and educational outcomes in sub-Saharan African countries, where evidence on this topic is limited.
- **Global health impact for policy and action:** Our findings should not discourage breastfeeding, as it is essential for infant health and nutrition.

## Background

Human breastmilk contains essential macronutrients and micronutrients that provide optimal nutrition for newborn growth and development [1–3]. It also contains many bioactive factors, including immunoglobulins, antimicrobial agents, and anti-inflammatory substances for newborn immunity programming against pathogens [1,4–6]. In addition to the short-term benefits of breastfeeding, some studies have suggested that optimally breastfed children might also benefit from improved educational outcomes [7–11]. While the protective effects of breastfeeding against pathogens have been established in both low-middle and high-income countries [12,13], the evidence supporting the suggested positive effects on educational outcomes is largely from high-income countries [9], where socioeconomic status strongly predicts both the pattern and duration of breastfeeding [14–16] and child educational outcomes [17,18].

It has been hypothesised that the positive effects reported are a manifestation of who breastfeeds in these populations and not a direct biological advantage of breastfeeding [19–22]. Indeed, in studies where socioeconomic confounders are controlled for, the strength of the association often attenuates substantially [10,23–25]. There are reasonable concerns that the small positive effects that often remain after accounting for socioeconomic status might still be biased by residual confounding from either imperfect measurement of these factors or inadequate adjustment [22,26,27]. In a 2002 systematic review with strict inclusion criteria, including a requirement for studies to control for socioeconomic status and stimulation of the child, there was no clear evidence of a positive effect of breastfeeding on intelligence when restricting to high-quality studies [22]. In a 2015 systematic review and meta-analysis that included studies mostly from high-income countries, breastfeeding was associated with higher scores in an intelligence test [28]. However, in a systematic review that included 13 studies from low- and middle-income countries, only five demonstrated a positive association between breastfeeding and cognitive development [21]. None of these reviews included studies from sub-Saharan Africa.

Evidence from sub-Saharan Africa could clarify this association since the duration and pattern of breastfeeding are not strongly influenced by socioeconomic status in the region [14,29,30]. Our recent systematic review revealed that only two studies had investigated the breastfeeding-educational-outcomes relationship in sub-Saharan Africa [31], with no studies from Uganda. Neither of the two South African studies identified in the review demonstrated a clear association between breastfeeding and educational outcomes [32,33], although our recent analysis of data from Malawi found better grade progression among children exclusively breastfed for a longer duration [34]. No clear evidence of association is found in other low- and middle-income countries, including Turkey, Guatemala, and the Philippines [32,35]. Yet, new evidence from high-income countries continues to show improved educational outcomes among children breastfed for a longer duration [36].

Given the discrepancies in the existing literature, further research is needed, particularly from sub-Saharan African countries, to understand the link between breastfeeding and educational outcomes. Sub-Saharan African countries also differ in childhood adversities, such as HIV exposure, which affect breastfeeding and the achievement of children [37]. The cohort analysed in this study includes mothers and children who are living with HIV [38] in rural Southwest Uganda. Residents of the study villages were followed over several years, and longitudinal data on feeding practices and schooling were collected at multiple time points. This study aimed to investigate whether the duration of breastfeeding in infancy was associated with educational attainment at primary school age using data from a large longitudinal population-based cohort in rural Southwest Uganda.

## Methods

We used longitudinal data from a population-based open cohort (the General Population Cohort) in rural Southwestern Uganda [38,39]. The cohort site is situated in Kalungu district, 120 km west of Kampala, the capital of Uganda, with a 2014 population of 183 232 [40]. Data on household members, including sociodemographic and housing characteristics, are collected through annual surveys [39]. Residents of the study villages, including children, are offered health care at the General Population Cohort (GPC) clinic located at the Kyamulibwa field station [38]. In 1999, child health surveys were introduced to collect detailed information about children under 13 years, including where they were born, feeding practices, vaccination status, anthropometry, and other child characteristics [39]. Trained field staff collected data using standard individual and household questionnaires moving from house to house [38,41]. Data collection was supervised by team leaders. Information across surveys was linked using unique participant, village, and household identification numbers issued to residents at their first participation. Details about the cohort, data collection, and management processes are published elsewhere [38,41,42]. For this analysis, the sample was restricted to children with information on breastfeeding and at least one primary school grade level measured by the 2005/2006 GPC survey, thereby only including children born between 1987 and 2000.

Retrospective information on breastfeeding practices was collected annually from mothers, including whether the mother ever breastfed the child, how many days after birth she began breastfeeding, the child’s current breastfeeding status, and the child’s age (in months) when the mother stopped breastfeeding. In the first round in which breastfeeding information was collected in 1999, mothers were asked about the breastfeeding of their older children. In subsequent rounds, breastfeeding information was collected from mothers of children aged 0–3 years.

In Uganda, primary school is compulsory and free in public schools, and children are expected to enter grade one at age six and advance from grade 1 to 7 in 7 years [43]. For example, children are expected to be in grade 4 at age 9, grade 5 at age 10, and grade 6 at age 11. At each annual survey, mothers or primary caregivers were asked if their child had ever enrolled in school and the child’s current grade level. We used this information together with the age of the child at the time of the survey to determine age-for-grade, defined as the expected grade level of a child at a given age if they started primary school at the official entry age without repeating or skipping a grade [34,44]. We then determined, at each age, whether a child was underage, on-time, or over-age for their current grade level for all time points for which schooling data were available for the child between ages six and 12. For example, for a 9-year-old, being in grade 3, was considered 1 year over-age, but grade 4 was considered on-time, and grade 5 one-year underage. Until 2005, promotion of primary school children to the next grade depended on performance, so age-for-grade is a marker of educational attainment. In 2005, automatic promotion was introduced [45] so only schooling data up to 2005 is included. Ethics approval for the present analysis was granted by the research ethics committee of the London School of Hygiene and Tropical Medicine (Ethics Ref: 26468).

## Data analysis

Baseline characteristics were summarised using percentages and frequencies. The duration of any breastfeeding was categorised as 0–6 months, 7–12 months, 13–17 months, 18–23 months, and >23 months. Frequencies and percentages were used to show the bivariate distribution of the participants’ characteristics across the breastfeeding groups. We followed UNESCO’s guidelines [46,47] to categorise children as underage for a grade if they were one or more years younger than the expected age for the grade, on-time if they were of the expected age or one year older than the expected age for the grade, and over-age for a grade if they were two or more years older than the expected age for the grade. We used graphs to illustrate the percentage of children underage, on-time, 2 years over-age, and 3 or more years over-age at each age from age 6 to 12.

Using binary logistic regression, we first examined the association between the duration of any breastfeeding and being 2 years, 3 or more years, or any years over-age for grade in primary school based on one age-for-grade attainment measured between ages 10 and 12. At these ages, children are expected to have completed the transition grade and be in upper primary. A child’s age-for-grade attainment at age 11 was first considered, and if this was not available, the attainment at age 12 was considered, and then at age 10 if there was no assessment for the child at age 12.

For age-for-grade attainment measured at multiple time points between ages six and 12, we used Generalised Estimation Equations (GEE) analysis with an exchangeable correlation structure to assess the association between the duration of any breastfeeding and being 2 years, 3 or more years, or any years over-age for grade between ages 8 and 12. We excluded age-for-grade assessments at ages 6 and 7 from the GEE analysis because no child was over-age for grade at these ages. The analysis accounted for the potential dependence of within-child repeated school measurements. We hypothesised that the effect of breastfeeding duration on educational attainment might differ depending on sex and age; therefore, in addition to the main analysis, we fitted separate GEE models with the repeated age-for-grade assessments for boys and girls, as well as for ages 8–9 and 10–12 years.

In the logistic regression and GEE analyses, we controlled for maternal education, maternal age, maternal HIV status, marital status, place of delivery, mode of delivery, household wealth, child sex, child year of birth, and survey year. Household wealth was estimated based on Principal Components Analysis (PCA) using data on ownership of assets (land, house, car, motorcycle, bicycle, telephone, radio, television, gas stove), dwelling characteristics (roof type, wall materials), livestock ownership, access to utilities (electricity and water), and whether the household employed a house help. There were different measures of household wealth across the survey rounds, with only a few rounds having some common indicators. For the PCA, categorical variables were reclassified as binary variables, and each survey round was analysed independently. For each round, the first component of the PCA was divided into quintiles ranging from lowest to highest household wealth and regrouped as low, middle, and high in the present analysis. Each mother-child dyad was assigned a household wealth that was calculated from indicators collected around the time of the child’s birth. Potential confounders were selected based on previous literature [9,31] and our knowledge of the relationship between breastfeeding and educational attainment.

Among the potential confounding variables, the percentage of missing values ranged from 0.4% for the mode of delivery to 40.5% for maternal education (Table 1). We used Little’s Missing Completely at Random (MCAR) test [48] to check the assumption that the data were MCAR and a chi-square test to examine the distribution of a missing indicator across the covariates. Other covariates predicted missingness and participants with complete data were systematically different from those with incomplete data. Additionally, Little’s MCAR test yielded a significant result (*p* < 0.001), suggesting that the data were not MCAR. The pattern of missingness revealed by these tests raised the possibility of biased results if the analyses were restricted to complete cases [49]. To reduce potential bias and loss of precision and power, we used Multiple Imputation by Chained Equations (MICE) to impute missing values in household wealth, place of delivery, mode of delivery, maternal education, and maternal age. Research on multiple imputation demonstrates that imputation can mitigate bias even in cases where the percentage of missing data is high [50]. We included all the variables in our substantive analyses models in the imputation model to ensure that the relationships between the variables of interest were preserved [49,51]. For each imputed model, 40 imputed datasets were generated based on the recommendation that the number of imputations should be at least equal to the proportion of missing observations [49,51]. Missing maternal HIV status was not imputed; ‘unknown’ was used as a third category to avoid excluding observations.

**Table 1.** Distribution of breastfeeding duration across the characteristics of the study participants.

		Duration of any breastfeeding				
	Total sample	0–6 months	7–12 months	13–17 months	18–23 months	>23 months
	n (%)	n (%)	n (%)	n (%)	n (%)	n (%)
All	3018	157 (5.2)	422 (14.0)	468 (15.5)	1059 (35.1)	912 (30.2)
**Child sex**						
Male	1584 (52.5)	71 (4.5)	208 (13.1)	259 (16.4)	553 (34.9)	493 (31.1)
Female	1434 (47.5)	86 (6.0)	214 (14.9)	209 (14.6)	506 (35.3)	419 (29.2)
**Child year of birth**						
1987 – 1989	290 (9.6)	20 (6.9)	45 (15.5)	47 (16.2)	105 (36.2)	73 (25.2)
1990 – 1994	1603 (53.1)	74 (4.6)	197 (12.3)	237 (14.8)	555 (34.6)	540 (33.7)
1995 – 2000	1125 (37.3)	63 (5.6)	180 (16.0)	184 (16.3)	399 (35.5)	299 (26.6)
**Mode of delivery**						
Vaginal	2955 (97.9)	146 (4.9)	413 (14.0)	459 (15.5)	1041 (35.2)	896 (30.3)
Surgical	50 (1.7)	5 (10.0)	7 (14.0)	8 (16.0)	15 (30.0)	15 (30.0)
Missing	13 (0.4)	6 (46.1)	2 (15.4)	1 (7.7)	3 (23.1)	1 (7.7)
**Place of delivery**						
Non-facility	1231 (40.8)	60 (4.9)	150 (12.2)	186 (15.1)	462 (37.5)	373 (30.3)
Facility	1744 (57.8)	86 (4.9)	265 (15.2)	278 (15.9)	584 (33.5)	531 (30.5)
Missing	43 (1.4)	11 (25.6)	7 (16.3)	4 (9.3)	13 (30.2)	8 (18.6)
**Maternal age**						
<20	351 (11.6)	26 (7.4)	62 (17.7)	66 (18.8)	113 (32.2)	84 (23.9)
20–29	1202 (39.8)	42 (3.5)	158 (13.1)	202 (16.8)	435 (36.2)	365 (30.4)
≥30	727 (24.1)	24 (3.3)	67 (9.2)	116 (16.0)	256 (35.2)	264 (36.3)
Missing	738 (24.5)	65 (8.8)	135 (18.3)	84 (11.4)	255 (34.5)	199 (27.0)
**Marital status**						
Unmarried	347 (11.5)	11 (3.2)	40 (11.5)	45 (13.0)	125 (36.0)	126 (36.3)
Married	1917 (63.5)	80 (4.2)	243 (12.7)	338 (17.6)	673 (35.1)	583 (30.4)
Missing	754 (25.0)	66 (8.8)	139 (18.4)	85 (11.3)	261 (34.6)	203 (26.9)
**Maternal education**						
None	49 (1.6)	1 (2.0)	2 (4.1)	9 (18.4)	14 (28.6)	23 (46.9)
Primary	1351 (44.8)	37 (2.7)	154 (11.4)	214 (15.9)	512 (37.9)	434 (32.1)
Secondary	352 (11.7)	18 (5.1)	60 (17.1)	72 (20.4)	107 (30.4)	95 (27.0)
Tertiary	45 (1.5)	2 (4.5)	9 (20.0)	11 (24.4)	10 (22.2)	13 (28.9)
Missing	1221 (40.5)	99 (8.1)	197 (16.1)	162 (13.3)	416 (34.1)	347 (28.4)
**Maternal HIV status**						
Positive	156 (5.2)	6 (3.9)	25 (16.0)	25 (16.0)	58 (37.2)	42 (26.9)
Negative	2123 (70.3)	86 (4.1)	262 (12.3)	359 (16.9)	745 (35.1)	671 (31.6)
Unknown	739 (24.5)	65 (8.8)	135 (18.3)	84 (11.4)	256 (34.6)	199 (26.9)
**Household wealth**						
Low	1060 (35.1)	61 (5.7)	144 (13.6)	151 (14.2)	378 (35.7)	326 (30.8)
Middle	577 (19.1)	21 (3.6)	87 (15.1)	100 (17.3)	207 (35.9)	162 (28.1)
High	1189 (39.4)	67 (5.6)	160 (13.5)	190 (16.0)	413 (34.7)	359 (30.2)
Missing	192 (6.4)	8 (4.2)	31 (16.1)	27 (14.1)	61 (31.8)	65 (33.8)

In addition to the main analysis with imputed data, we performed a sensitivity analysis that only included participants with complete data. In the complete case analysis, we fitted both the binary logistic regression and the GEE models for the total sample. Due to the small sample size in the subgroups, the complete case analysis did not include age and gender subgroup analyses.

## Results

### Characteristics of the study sample

The analytic cohort consisted of 3018 children for whom breastfeeding information was available and who were followed for at least 5 years, with at least one primary school grade recorded by the 2005 to 2006 survey. Table 1 presents the characteristics of the study sample. Nearly all the children (97.9%) were delivered through vaginal birth, and 57.8% of the births occurred in a healthcare facility. Just over half of the children were male (52.5%), born between 1990 and 1994 (53.1%), and 39.8% of the mothers were between the ages of 20 and 29 when their children were born. The majority (70.3%) of mothers were known to be HIV negative; 5.2% tested positive. Maternal education was mostly primary (44.8%), with only 1.5% having a tertiary education.

### Breastfeeding duration

Of 3018 children, 5.2% were breastfed for less than 7 months, 14.0% for 7 to 12 months, and 15.5% for 13–17 months (Table 1). A little over one-third of mothers (35.1%) breastfed for 18–23 months, with 30.2% breastfeeding for more than 23 months. There was no considerable difference in the duration of breastfeeding by child sex, delivery mode, place of birth, or level of household wealth. However, older mothers were more likely to breastfeed for a longer duration than younger mothers, and a higher percentage of mothers with no education and those with primary education breastfed for 2 years or longer than those with post-primary education. Mothers who tested negative for HIV were more likely to breastfeed for 2 years or more than those who tested positive.

### Age-for-grade attainment

**Figure 1.**
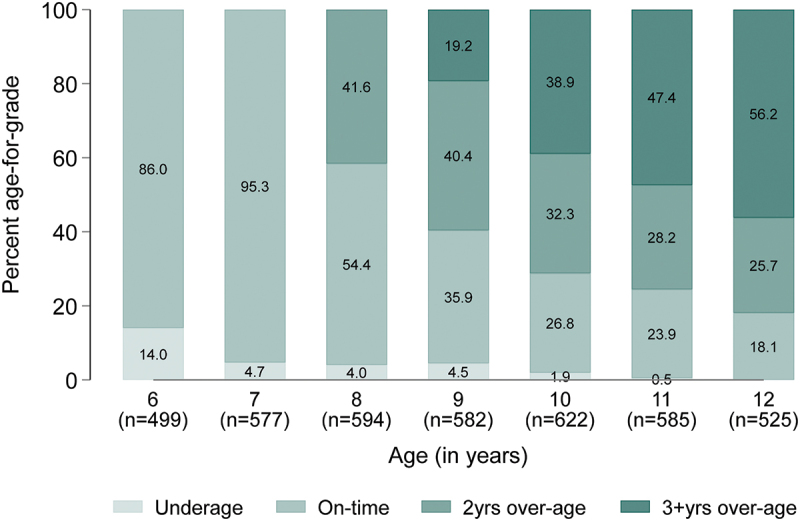
Distribution of age-for-grade by child’s age at school assessment (Underage = one or more years younger than the expected age for a grade; On-time = at the expected age for a grade or 1 year older than the expected age for a grade; 2 years over-age = 2 years older than the expected age for a grade; and 3+years over-age = 3 or more years older than the expected age for a grade for a grade).

**Figure 2.**
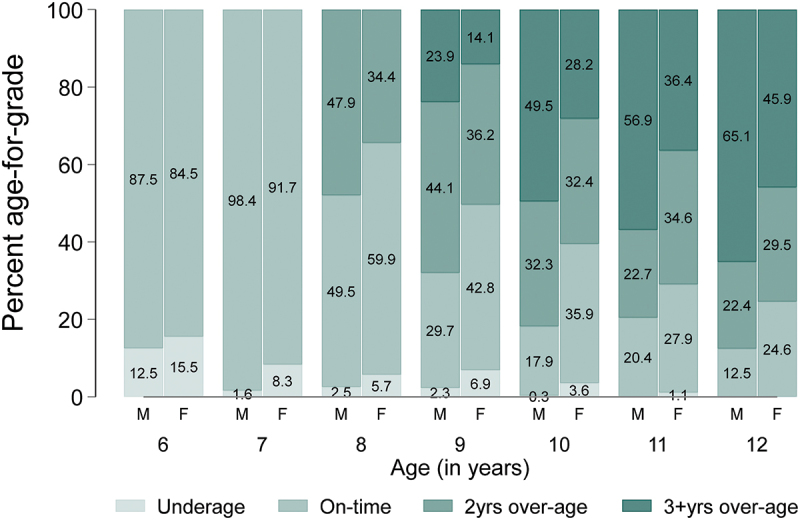
Distribution of age-for-grade at each age by child’s sex (Underage = one or more years younger than the expected age for a grade; On-time = at the expected age for a grade or one year older than the expected age for a grade; 2 years over-age = 2 years older than the expected age for a grade; and 3+years over-age = 3 or more years older than the expected age for a grade for a grade. [M = male F = Female]).

At age six, data on age-for-grade were available for 499 children (Figure 1). This number increased to 622 at age 10 before declining to 525 at age 12. The percentage of children underage for grade at each age declined from 14.0% at age six to 0.5% at age 11, with no child underage for grade at age 12. The percentage on-time for grade at each age also declined steadily from 86.0% at age 6 to 18.1% at age 12. However, by age 8, 41.6% of the children were 2 years older than the appropriate age for their grade level, but this percentage dropped to 25.7% at age 12. In contrast, the percentage 3 or more years older than the appropriate age for their grade steadily increased from 19.2% at age 9 to 56.2% at age 12 (Figure 1). At each age, information on age-for-grade was available for more boys than girls. However, a higher percentage of girls than boys were underage for grade from age 6 to 11, and girls were also less likely to be over-age for grade than boys (Figure 2).

### Association between duration of any breastfeeding and being over-age for grade based on a single age-for-grade assessment between ages 10 and 12 years

Table 2 presents the unadjusted and adjusted odds ratios for the association between the duration of any breastfeeding and being 2 years, 3 or more years, or any years over-age for grade in primary school based on assessment at one point between ages 10 and 12. In the unadjusted analysis, the odds ratios for being 2 years, three or more years, or any years over-age for grade among children breastfed for 7–12, 13–17, 18–23, and >23 months were lower compared to those breastfed for 0–6 months, except in the 18–23 months breastfeeding group (OR 1.02, 95%CI 0.49–2.11) for 2 years over-age for grade. After adjusting for confounding factors, the odds ratios strengthened for all breastfeeding categories, including a decrease in the 18–23 months breastfeeding group (aOR 0.95, 95%CI 0.43–2.07) for 2 years over-age for grade. The odds of being 2 years, 3 or more years, or any years over-age for grade among children breastfed for 7–12, 13–17, 18–23, and >23 months were lower than those breastfed for 0–6 months. However, there was no evidence of an overall association between breastfeeding duration and over-age for grade (2 years: *p* = 0.82; three or more years: *p* = 0.50; and any over-age: *p* = 0.77).

**Table 2.** Binary logistic regression analysis of the association between duration of breastfeeding and being over-age for grade at one point between ages 10 and 12 in Uganda.

	Two years over-age for grade vs on-time for grade		Three or more years over-age for grade vs on-time for grade		Over-age for grade vs on-time for grade	
	OR (95% CI)	aOR (95% CI)	OR (95% CI)	aOR (95% CI)	OR (95% CI)	aOR (95% CI)
**Model 1: Both sexes**						
**Duration of any breastfeeding**	*n* = 797		*n* = 1076		*n* = 1516	
	*p* = 0.73	*p* = 0.82	*p* = 0.89	*p* = 0.50	*p* = 0.86	*p* = 0.77
0–6 months	1.00	1.00	1.00	1.00	1.00	1.00
7–12 months	0.78 (0.35–1.73)	0.77 (0.33–1.79)	0.69 (0.34–1.40)	0.60 (0.27–1.36)	0.72 (0.37–1.41)	0.69 (0.33–1.41)
13–17 months	0.90 (0.41–1.96)	0.87 (0.38–2.00)	0.71 (0.35–1.42)	0.53 (0.24–1.18)	0.77 (0.40–1.50)	0.68 (0.33–1.37)
18–23 months	1.02 (0.49–2.11)	0.95 (0.43–2.07)	0.75 (0.39–1.43)	0.51 (0.24–1.08)	0.84 (0.45–1.55)	0.68 (0.35–1.33)
>23 months	0.84 (0.40–1.74)	0.78 (0.36–1.71)	0.74 (0.39–1.42)	0.55 (0.26–1.15)	0.77 (0.42–1.44)	0.65 (0.33–1.27)
**Model 2: Boys**						
**Duration of any breastfeeding**	*n* = 336		*n* = 591		*n* = 796	
	*p* = 0.28	*p* = 0.31	*p* = 0.35	*p* = 0.56	*p* = 0.26	*p* = 0.43
0–6 months	1.00	1.00	1.00	1.00	1.00	1.00
7–12 months	0.29 (0.07–1.19)	0.26 (0.06–1.20)	0.33 (0.09–1.24)	0.31 (0.08–1.26)	0.32 (0.09–1.14)	0.29 (0.07–1.11)
13–17 months	0.52 (0.13–2.13)	0.47 (0.10–2.14)	0.56 (0.15–2.09)	0.43 (0.11–1.75)	0.55 (0.15–1.98)	0.42 (0.11–1.62)
18–23 months	0.55 (0.14–2.11)	0.53 (0.13–2.28)	0.56 (0.16–1.98)	0.44 (0.12–1.69)	0.56 (0.16–1.91)	0.42 (0.11–1.53)
>23 months	0.42 (0.11–1.63)	0.40 (0.09–1.75)	0.54 (0.15–1.91)	0.41 (0.11–1.58)	0.50 (0.15–1.72)	0.38 (0.10–1.39)
**Model 3: Girls**						
**Duration of any breastfeeding**	*n *= 461		*n* = 485		*n* = 720	
	*p* = 0.89	*p* = 0.85	*p* = 0.50	*p* = 0.39	*p* = 0.78	*p* = 0.79
0–6 months	1.00	1.00	1.00	1.00	1.00	1.00
7–12 months	1.41 (0.51–3.93)	1.60 (0.51–4.97)	0.96 (0.39–2.36)	1.04 (0.37–2.97)	1.11 (0.48–2.56)	1.20 (0.48–3.01)
13–17 months	1.15 (0.42–3.14)	1.31 (0.44–3.91)	0.58 (0.24–1.40)	0.60 (0.21–1.68)	0.77 (0.34–1.73)	0.88 (0.36–2.14)
18–23 months	1.39 (0.55–3.53)	1.41 (0.50–3.98)	0.67 (0.30–1.50)	0.57 (0.22–1.47)	0.91 (0.43–1.92)	0.85 (0.37–1.95)
>23 months	1.19 (0.47–3.04)	1.17 (0.42–3.31)	0.66 (0.29–1.49)	0.64 (0.25–1.66)	0.84 (0.39–1.78)	0.82 (0.35–1.88)

We controlled for maternal education, household wealth, maternal age, maternal HIV status, marital status, place of delivery, mode of delivery, child sex, child year of birth, and survey year.

A similar pattern emerged in the unadjusted and adjusted subgroup analyses for boys. Those breastfed for 7–12, 13–17, 18–23, and >23 months had lower odds of being 2 years, 3 or more years, or any years over-age for grade compared to those breastfed for 0–6 months, but there was no evidence to support an overall association (2 years over-age: *p* = 0.31; 3 or more years over-age: *p* = 0.56; and any over-age: *p* = 0.43).

For girls, the adjusted and unadjusted odds ratios were suggestive of higher odds for 2 years over-age for grade in all breastfeeding duration categories (7–12 months [aOR 1.60, 95%CI 0.51–4.97]; 13–17 months [aOR 1.31, 95%CI 0.44–3.91]; 18–23 months: [aOR 1.41, 95%CI 0.50–3.98]; and >23 months [aOR 1.17 95%CI 0.42–3.31]) compared to those breastfed for 0–6 months, although there was no evidence for an overall association (*p* = 0.85). The odds of being 3 or more years over-age or any over-age for grade among girls were lower in all breastfeeding groups than breastfeeding for 0–6 months after adjustment except in the 7–12 months breastfeeding groups, but there was no evidence for an association.

Results of the complete case analysis involving only the small number of participants with complete observations for all variables differed slightly in the direction of association. However, similar to the main analysis, there was no evidence to support an overall association between breastfeeding duration and over-age for grade (Supplementary Table S1).

### Association between duration of any breastfeeding and being over-age for grade based on repeated age-for-grade assessments from ages 8 to 12

Table 3 shows the unadjusted and adjusted odds ratios for the association between the duration of any breastfeeding and being 2 years, 3 or more years, or any years over-age for grade in primary school based on repeated age-for-grade assessments between ages 8 and 12. In the total sample, the unadjusted results were generally consistent with reduced odds of being 2 years, 3 or more years, or any years over-age for grade among children breastfed for 7–12, 13–17, 18–23, and >23 months compared to those breastfed for 0–6 months, though there was no evidence of association. After adjusting for confounding factors, the odds ratios for almost all breastfeeding duration groups strengthened, and the odds of being 2 years, 3 or more years, or any years over-age for grade were lower in all the breastfeeding groups compared to breastfeeding for 0–6 months. However, there was no strong evidence to support an overall association between the duration of any breastfeeding and being 2 years (*p* = 0.99), 3 or more years (*p* = 0.48), or any years over-age (*p* = 0.85) for grade.

**Table 3.** Generalised estimating equations analysis of the association between breastfeeding duration and being over-age for grade in primary school among children aged 8–12 in Uganda.

	Two years over-age for grade vs on-time for grade		Three or more years over-age for grade vs on-time for grade		Over-age for grade vs on-time for grade	
	OR (95% CI)	aOR (95% CI)	OR (95% CI)	aOR (95% CI)	OR (95% CI)	aOR (95% CI)
**Model 1: Both sexes at ages 8–12**						
**Duration of any breastfeeding**	*n* = 1729		*n *= 1668		*n *= 2368	
	*P *= 0.97	*P *= 0.99	*P *= 0.75	*P *= 0.48	*P *= 0.86	*P *= 0.85
0–6 months	1.00	1.00	1.00	1.00	1.00	1.00
7–12 months	0.92 (0.56–1.54)	0.95 (0.55–1.66)	0.74 (0.45–1.22)	0.68 (0.38–1.20)	0.82 (0.53–1.27)	0.85 (0.52–1.39)
13–17 months	0.93 (0.56–1.54)	0.91 (0.52–1.58)	0.77 (0.47–1.27)	0.61 (0.35–1.07)	0.83 (0.54–1.28)	0.76 (0.47–1.24)
18–23 months	1.01 (0.63–1.62)	0.98 (0.58–1.66)	0.82 (0.52–1.30)	0.65 (0.38–1.09)	0.90 (0.60–1.35)	0.81 (0.51–1.29)
>23 months	0.98 (0.61–1.58)	0.95 (0.56–1.62)	0.85 (0.54–1.35)	0.62 (0.37–1.06)	0.89 (0.59–1.35)	0.80 (0.50–1.28)
**Model 2: Boys aged 8–12**						
**Duration of any breastfeeding**	*n *= 830		*n *= 877		*n *= 1245	
	*P *= 0.43	*P *= 0.60	*P *= 0.35	*P *= 0.75	*P *= 0.33	*P *= 0.64
0–6 months	1.00	1.00	1.00	1.00	1.00	1.00
7–12 months	1.02 (0.47–2.21)	0.93 (0.39–2.18)	0.66 (0.33–1.34)	0.53 (0.21–1.33)	0.76 (0.40–1.45)	0.71 (0.35–1.43)
13–17 months	1.38 (0.65–2.94)	1.26 (0.55–2.92)	0.88 (0.44–1.75)	0.65 (0.26–1.63)	1.01 (0.54–1.89)	0.93 (0.47–1.85)
18–23 months	1.42 (0.69–2.93)	1.26 (0.56–2.83)	0.96 (0.50–1.83)	0.64 (0.27–1.51)	1.08 (0.60–1.97)	0.94 (0.49–1.80)
>23 months	1.49 (0.72–3.06)	1.34 (0.60–3.01)	1.02 (0.54–1.96)	0.65 (0.28–1.53)	1.14 (0.63–2.07)	0.99 (0.51–1.90)
**Model 3: Girls aged 8–12**						
**Duration of any breastfeeding**	*n *= 899		*n *= 791		*n *= 1123	
	*P *= 0.46	*P *= 0.52	*P *= 0.49	*P *= 0.30	*P *= 0.37	*P *= 0.29
0–6 months	1.00	1.00	1.00	1.00	1.00	1.00
7–12 months	0.86 (0.44–1.69)	1.08 (0.51–2.29)	0.84 (0.41–1.73)	1.11 (0.50–2.45)	0.86 (0.47–1.59)	1.06 (0.54–2.09)
13–17 months	0.62 (0.31–1.22)	0.78 (0.36–1.65)	0.58 (0.28–1.21)	0.70 (0.31–1.60)	0.62 (0.33–1.14)	0.73 (0.37–1.44)
18–23 months	0.76 (0.41–1.42)	0.86 (0.42–1.74)	0.68 (0.35–1.32)	0.74 (0.34–1.58)	0.73 (0.42–1.29)	0.78 (0.41–1.49)
>23 months	0.67 (0.36–1.26)	0.75 (0.37–1.52)	0.66 (0.34–1.30)	0.68 (0.31–1.47)	0.68 (0.39–1.20)	071 (0.37–1.35)
**Model 4: Both sexes at ages 8 and 9**						
**Duration of any breastfeeding**	*n *= 1043		*n *= 683		*n *= 1151	
	*P *= 0.95	*P *= 0.93	*P *= 0.89	*P *= 0.91	*P *= 0.96	*P *= 0.97
0–6 months	1.00	1.00	1.00	1.00	1.00	1.00
7–12 months	1.05 (0.53–2.05)	1.09 (0.51–2.34)	1.64 (0.44–6.11)	1.41 (0.34–5.83)	1.13 (0.59–2.14)	1.11 (0.53–2.31)
13–17 months	0.96 (0.49–1.88)	0.98 (0.45–2.13)	1.52 (0.41–5.65)	1.15 (0.28–4.69)	1.03 (0.54–1.96)	0.96 (0.46–2.03)
18–23 months	1.00 (0.53–1.87)	1.01 (0.49–2.09)	1.81 (0.52–6.26)	1.29 (0.35–4.72)	1.10 (0.60–2.01)	1.02 (0.51–2.05)
>23 months	1.10 (0.59–2.07)	1.14 (0.55–2.36)	1.55 (0.44–5.45)	1.01 (0.28–3.73)	1.16 (0.64–2.13)	1.07 (0.53–2.18)
**Model 5: Both sexes at ages 10–12**						
**Duration of any breastfeeding**	*n *= 876		*n *= 1167		*n *= 1626	
	*P *= 0.82	*P *= 0.82	*P *= 0.88	*P *= 0.55	*P *= 0.87	*P *= 0.81
0–6 months	1.00	1.00	1.00	1.00	1.00	1.00
7–12 months	0.87 (0.41–1.82)	0.85 (0.37–1.93)	0.71 (0.37–1.39)	0.75 (0.35–1.62)	0.75 (0.40–1.41)	0.84 (0.42–1.67)
13–17 months	0.87 (0.42–1.80)	0.83 (0.37–1.86)	0.71 (0.37–1.37)	0.62 (0.29–1.32)	0.74 (0.40–1.38)	0.71 (0.36–1.41)
18–23 months	1.01 (0.51–1.98)	0.93 (0.43–2.02)	0.74 (0.40–1.36)	0.58 (0.28–1.19)	0.81 (0.46–1.44)	0.73 (0.38–1.40)
>23 months	0.83 (0.42–1.65)	0.77 (0.35–1.65)	0.74 (0.40–1.37)	0.64 (0.31–1.30)	0.76 (0.43–1.35)	0.71 (0.37–1.36)

We controlled for maternal education, household wealth, maternal age, maternal HIV status, marital status, place of delivery, mode of delivery, child sex, child year of birth, and survey year.

In the unadjusted sex-stratified analysis, the odds ratios for boys breastfed for 7–12 months (OR = 1.02, 95%CI = 0.47–2.21), 13–17 months (OR = 1.38, 95%CI = 0.65–2.94), 18–23 months (OR = 1.42, 95%CI = 0.69–2.93), and >23 months (OR = 1.49, 95%CI = 0.72–3.06) were consistent with being more likely to be 2 years over-age for grade than those breastfed for 0–6 months (p-value = 0.43). The odds ratios weakened slightly after adjustment (7–12 months: OR 0.93, 95%CI 0.39–2.18; 13–17 months: OR 1.26, 95%CI 0.55–2.92; 18–23 months: OR 1.26, 95%CI 0.56–2.83; and >23 months: OR 1.34, 95%CI 0.60–3.01) but there was no evidence to support a difference in the odds of being 2 years over-age for grade among boys breastfed for varying durations (p-value = 0.60). The odds ratios for the association between breastfeeding duration and being 3 or more years or any years over-age for grade among boys strengthened after adjusting for confounding factors and were lower in all breastfeeding groups compared to breastfeeding for 0–6 months. However, there was no evidence of an association between breastfeeding duration and being 3 years or any over-age for grade among boys.

The odds of being 2 years, 3 years, or any years over-age for grade were lower in all breastfeeding duration groups than breastfeeding for 0–6 months in both the unadjusted and adjusted sex-stratified analysis for girls, except in the 7–12 months group. However, there was no evidence to support an overall association between the duration of breastfeeding and being 2 years (*p*-value = 0.52), 3 or more years (*P*-value = 0.30), or any years over-age (*P*-value = 0.29) for grade among girls.

In the age-stratified analysis, similar patterns emerged in the association between breastfeeding duration and being 2 years, 3 or more years, or any years over-age for grade, with odds ratios either attenuating or strengthening after adjustment. However, there was no evidence of an association between breastfeeding duration and being over-age for grade in the age subgroups.

Results of the complete case analysis involving only the small number of participants with complete observations for all variables differed slightly. However, similar to the main analysis, there was no evidence to support an overall association between breastfeeding duration and over-age for grade (Supplementary Table S2).

## Discussion

We used population-based cohort data to examine the association between breastfeeding duration and educational attainment in Uganda. Breastfeeding duration in infancy was not associated with age-for-grade attainment during the school-age years. These findings remained consistent when we stratified the sample by sex and age. Despite the lack of evidence for an association, the data showed a consistent trend of lower odds of being over-aged for grade with a longer duration of breastfeeding after controlling for confounders.

Our results are consistent with existing literature on the association of breastfeeding with educational outcomes in sub-Saharan Africa [31]. For example, an analysis of data from the birth-to-twenty cohort study in South Africa found no effect of breastfeeding duration in infancy on subsequent educational outcomes among 17-year-olds [32]. Similarly, when Mitchell et al. [33] studied 7–11-year-olds in rural KwaZulu-Natal, South Africa, they found no conclusive evidence of an association between exclusive breastfeeding and grade repetition. In addition, while our recent analysis of data from a Malawian cohort suggested an association between exclusive breastfeeding and age-for-grade attainment [34], in our earlier systematic review of data from sub-Saharan Africa, we found no effect of breastfeeding on cognitive development or educational achievement [31].

Various interconnected factors influence educational attainment in sub-Saharan Africa, including cultural and environmental factors, educational quality, and family support [52,53]. Breastfeeding, while undoubtedly beneficial for infant health and development, may have a limited direct influence on educational outcomes [19,26,32,33]. Studies that found better educational outcomes among optimally breastfed children compared to those with suboptimal breastfeeding, mostly from high-income countries, suggest that this benefit could be attributed to the physiological effect of breastmilk on cognitive development and intelligence [7,8,11]. However, given that the actual effects of breastfeeding on cognitive development and performance in intelligence tests are modest [23,54,55], it has been argued that these small effects are unlikely to translate into real-world improvements in educational achievement. Although a cluster-randomised Breastfeeding Promotion Intervention Trial in Belarus found a positive effect of breastfeeding on cognitive development [56], a similar large cluster-randomised controlled trial of breastfeeding promotion that markedly increased exclusive breastfeeding in the intervention group in Uganda and Burkina Faso found no effect of exclusive breastfeeding on cognitive development [57,58].

Residual socioeconomic confounding could account for the reported positive effects of breastfeeding on educational outcomes in predominantly high-income settings. In this study, breastfeeding was not associated with household income, and women with higher education were not more likely to breastfeed. However, in high-income countries, socioeconomic status has a positive impact on breastfeeding patterns and duration. For example, in a study that found better educational outcomes among breastfed children in the United States, mothers of breastfed children were more likely to be employed, have a higher education, and have fewer financial problems [9]. In a recent analysis of data from the Millennium Cohort Study in England, mothers who breastfed for a longer duration were more likely to be educated and of a higher social class [36]. Similar socioeconomic inequality in breastfeeding was found in a New Zealand study sample with higher mean test scores among children who were breastfed [25]. Although these studies account for some measures of socioeconomic status, residual confounding from unobserved or inaccurate measurements of these factors cannot be ruled out.

It is also possible that the discrepancy between our results and those of previous studies that found evidence of a positive association is attributable to differences in the breastfeeding duration groups compared. In this Ugandan cohort, all the children received breastmilk, albeit with varying durations of breastfeeding, with only about 1.0% breastfed for less than a month. However, in most studies that reported a positive association, the effect of breastfeeding duration on educational outcomes among breastfed children is often compared with non-breastfed children [7,9,10,36,59]. In this analysis, there was no suitable data to investigate the impact of exclusive breastfeeding on attainment or compare the breastfeeding groups to those never breastfed.

Even in high-income countries, where the majority of studies report a positive effect of breastfeeding on educational outcomes, there are some inconsistencies. For example, in a study among 10-year-olds in Australia, the duration of any breastfeeding was not associated with educational outcomes after adjustments, though predominant breastfeeding was associated with higher academic scores [11]. Similarly, when researchers examined the effect of breastfeeding duration on reading and math test scores in American children, they found no significant difference in test scores among the breastfeeding duration groups [59].

Breastfeeding has numerous established health benefits for both the mother and the infant [60,61]. Even though this study did not find evidence of an association between breastfeeding duration and age-for-grade attainment, our findings should not discourage breastfeeding practices, as breastfeeding plays an essential role in infant nutrition, immunity, and overall well-being [1,4–6].

An important strength of our study is its longitudinal design, which allowed us to assess educational attainment over time. Also, the sample size was relatively large. The main limitation is the use of retrospective breastfeeding data, which is susceptible to recall bias and social desirability bias, which could result in overreporting. Furthermore, the lack of information on exclusive breastfeeding limits the generalizability of the results. In addition, while efforts were made to control for potential confounding variables, residual confounding cannot be ruled out, and we did not adjust for birthweight, paternal education, and maternal intelligence. However, adjusting for these variables is unlikely to change the conclusion of our findings since it has been shown that they tend to reduce the magnitude of the effect and the strength of the association [7,8,36].

The use of age-for-grade as a measure of educational attainment has some limitations. It does not account for factors such as grade repetition due to missed schooling or other unique circumstances. Children from disadvantaged backgrounds might face unobserved obstacles that affect their grade progression, even if they have high academic potential. However, because grade progression in Uganda pre-2005 was largely based on classroom performance, any potential bias is likely minimal. Our findings should be interpreted cautiously, as the total and subgroup analyses were underpowered. Some children were clustered at the level of the mothers, but because over 40% of children were missing maternal identification numbers, we did not include this in our analysis models. Although the data for this analysis were not recent, it is unlikely that the association would change over time if there were a biological explanation for it. Our findings are generalisable to similar Ugandan populations, given comparable country-level breastfeeding and schooling practices and breastmilk composition among women. However, caution should be exercised in generalising to other sub-Saharan African settings.

## Conclusions

In this cohort, most mothers breastfed for a longer duration, suggesting widespread cultural acceptance of breastfeeding in Uganda. Although we found no association between breastfeeding duration and educational attainment, breastfeeding remains an important component of early childhood health and nutrition. Mothers who are able to breastfeed should be encouraged and supported to initiate and continue breastfeeding for at least 2 years after childbirth. Future research could explore the effects of exclusive breastfeeding and use various measures of educational attainment. Additionally, studies in different sub-Saharan African countries could contribute to a more comprehensive understanding of the relationship between breastfeeding and educational outcomes and whether it has a biological effect on achievement.

## Supplementary Material

Supplemental Material
