# Supplementary material for: Association between breastfeeding duration and educational attainment in rural Southwest Uganda: a population-based cohort study
Source: Glob Health Action. 2024 Apr 23;17(1):2338023. doi: 10.1080/16549716.2024.2338023 (PMC11041517; doi:10.1080/16549716.2024.2338023)
Supplement: Supplemental Material [file ZGHA_A_2338023_SM7752.docx]

**Supplementary File**

**Supplementary Table 1 (complete case analysis): Binary logistic regression analysis of the association between duration of any breastfeeding and over-age for grade at one point between ages 10 and 12 in Uganda**

|  | **Two years over-age for grade vs**  **on-time for grade** | |  | **Three or more years over-age for grade vs on-time for grade** | |  | **Over-age for grade vs**  **on-time for grade** | |
| --- | --- | --- | --- | --- | --- | --- | --- | --- |
|  | **OR (95% CI)** | **aOR (95% CI)** |  | **OR (95% CI)** | **aOR (95% CI)** |  | **OR (95% CI)** | **aOR (95% CI)** |
| **Model 1: Both sexes at ages 10-12** | |  |  |  |  |  |  |  |
| **Duration of any breastfeeding** | n=445 | |  | n = 574 | |  | n = 819 | |
|  | P = 0.22 | P = 0.67 |  | P = 0.56 | P = 0.46 |  | P = 0.54 | P = 0.83 |
| 0-6 months | 1.00 | 1.00 |  | 1.00 | 1.00 |  | 1.00 | 1.00 |
| 7-12 months | 0.88 (0.27 - 2.83) | 0.98 (0.28 - 3.39) |  | 1.37 (0.47 - 4.00) | 1.75 (0.51 - 5.93) |  | 1.15 (0.44 - 3.03) | 1.48 (0.52 - 4.21) |
| 13-17 months | 1.37 (0.45 - 4.16) | 1.15 (0.36 - 3.68) |  | 1.55 (0.55 - 4.38) | 1.52 (0.47 - 4.92) |  | 1.47 (0.58 - 3.73) | 1.47 (0.54 - 4.00) |
| 18-23 months | 1.78 (0.62 - 5.14) | 1.53 (0.50 - 4.65) |  | 1.62 (0.60 - 4.39) | 1.48 (0.48 - 4.57) |  | 1.69 (0.69 - 4.12) | 1.57 (0.60 - 4.10) |
| >23 months | 1.27 (0.44 - 3.70) | 1.16 (0.37 - 3.59) |  | 1.98 (0.73 - 5.36) | 2.14 (0.69 - 6.68) |  | 1.67 (0.68 - 4.08) | 1.74 (0.66 - 4.56) |

Note: We controlled for maternal education, household wealth, maternal age, maternal HIV status, marital status, place of delivery, mode of delivery, child sex, child year of birth, and survey year.

**Supplementary Table 2 (Complete case analysis): Generalised estimating equations analysis of the association between breastfeeding duration and being over-age for grade in primary school among children aged 8–12 in Uganda**

|  | **Two years over-age for grade vs**  **on-time for grade** | |  | **Three or more years over-age for grade vs on-time for grade** | |  | **Over-age for grade vs**  **on-time for grade** | |
| --- | --- | --- | --- | --- | --- | --- | --- | --- |
|  | **OR (95% CI)** | **aOR (95% CI)** |  | **OR (95% CI)** | **aOR (95% CI)** |  | **OR (95% CI)** | **aOR (95% CI)** |
| **Model 1: Both sexes at ages 8-12** | |  |  |  |  |  |  |  |
| **Duration of any breastfeeding** | n = 987 | |  | 932 | |  | n = 1323 | |
|  | P = 0.36 | P = 0.42 |  | P = 0.25 | P = 0.31 |  | P = 0.22 | P = 0.25 |
| 0-6 months | 1.00 | 1.00 |  | 1.00 | 1.00 |  | 1.00 | 1.00 |
| 7-12 months | 1.14 (0.50 - 2.61) | 1.15 (0.46 - 2.86) |  | 1.36 (0.57 - 3.24) | 1.86 (0.77 - 4.50) |  | 1.22 (0.61 - 2.47) | 1.42 (0.65 - 3.13) |
| 13-17 months | 1.24 (0.56 - 2.76) | 1.12 (0.47 - 2.68) |  | 1.33 (0.57 - 3.09) | 1.45 (0.61 - 3.41) |  | 1.26 (0.64 - 2.49) | 1.20 (0.56 - 2.56) |
| 18-23 months | 1.48 (0.69 - 3.20) | 1.36 (0.58 - 3.19) |  | 1.39 (0.61 - 3.14) | 1.50 (0.66 - 3.37) |  | 1.43 (0.75 - 2.76) | 1.42 (0.68 - 2.96) |
| >23 months | 1.59 (0.74 - 3.43) | 1.53 (0.65 - 3.59) |  | 1.81 (0.80 - 4.09) | 1.95 (0.87 - 4.41) |  | 1.67 (0.87 - 3.22) | 1.70 (0.81 - 3.57) |

Note: We controlled for maternal education, household wealth, maternal age, maternal HIV status, marital status, place of delivery, mode of delivery, child sex, child year of birth, and survey year.
